# Patterning of Photochromic Diarylethene Crystals by Sublimation for Morphological Controls

**DOI:** 10.1002/smtd.202401545

**Published:** 2025-01-19

**Authors:** Mami Isobe, Daichi Kitagawa, Seiya Kobatake

**Affiliations:** ^1^ Department of Chemistry and Bioengineering Graduate School of Engineering Osaka Metropolitan University 3‐3‐138 Sugimoto, Sumiyoshi‐ku Osaka 558‐8585 Japan

**Keywords:** crystal growth, diarylethene, patterning, photochromism, sublimation

## Abstract

A patterned growth of crystals of 1,2‐bis(2,5‐dimethyl‐3‐thienyl)perfluorocyclopentene (**1a**) on the glass substrate with convex guides is reported by sublimation methods. The lower supersaturation of substrate surfaces with higher temperatures can facilitate the vapor‐to‐liquid process rather than the vapor‐to‐crystal process in the early stage of the sublimation. Micro‐droplets of melts of **1a** are generated on the sidewalls of the convex guides, then crystallized into the microcrystals, accompanied by the rearrangements of the crystallographic in‐plane orientations. Moreover, the crystalline patterns fringed with the rod crystals are colored red upon irradiation with ultraviolet light. This well‐controllability of crystal morphologies in a simple use of sublimation methods will pave the way for large‐sized photomechanical materials with the desired morphologies.

## Graphical abstract

Substrate temperature is used for controlling generations of crystalline and liquid phases of sublimates. A patterned crystal growth of 1,2‐bis(2,5‐dimethyl‐3‐thienyl)perfluorocyclopentene (**1a**) stems from crystallizations of melts of **1a** generated on sidewalls of convex guides on substrates with a certain temperature. Crystalline patterns having the shape of “18” finally appear through such crystallizations led by the convex guide shaped into “18”.

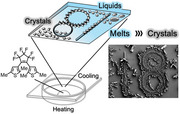

## 1 Introduction

Diarylethene is one of the photochromic compounds expected to exhibit reversible photomechanical behaviors, and photo‐induced crystal shape changes, in the solid states under alternating irradiation with ultraviolet (UV) and visible (vis.) light.^[^ ^1^ ^]^ Crystal morphologies such as rod and ribbon‐shaped crystals can affect the photomechanical bending^[^ ^2^ ^]^ and twisting.^[^ ^3^ ^]^ In the fabrication of such photomechanical crystals, the crystallographic growth orientations, associated with the crystal morphologies, require controlling precisely in the preparation process. The morphological control of these diarylethene crystals has been challenging for the conventional simple methods such as slow evaporation, which cannot meet the demand for sufficient management of thermodynamic conditions.

Patterned growth of organic crystals has greatly contributed to the development and improvement of well‐defined geometric features for organic field‐effect transistors.^[^ ^4^ ^]^ In particular, the patterning based on vapor deposition methods^[^ ^5^ ^]^ may be highly appreciable to morphological designs of diarylethene crystals through the positioning of crystallizations and the tailoring of crystallographic growth directions on substrates. Therefore, it is essential to incorporate the vapor phase crystal growth methodologies for diarylethene derivatives into the patterning techniques for organic materials.

**Figure 1:**
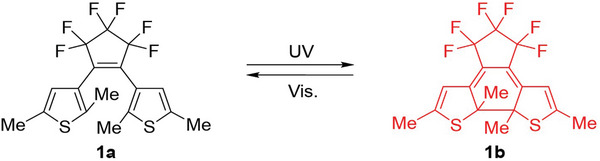
Photochromism of **1a**.

In our studies, sublimations of 1,2‐bis(2,5‐dimethyl‐3‐thienyl)perfluorocyclopentene (**1a**) (**Figure** **1**) on the glass substrates have been conducted under an atmospheric environment.^[^ ^6^ ^]^ The crystal morphologies of polycrystalline thin films of **1a** grown on the substrate surface can be controlled by its wettability. Rod crystals with different growth directions are subsequently generated on these thin films.^[^ ^6a^ ^]^ The hyperbranched rod crystals of **1a** are finally produced on the spherical substrate with a large curvature.^[^ ^6b^ ^]^ These sublimation techniques have taken on the controllability for crystal morphologies ahead of other in‐air sublimation methodologies.^[^ ^7^ ^]^ On the other hand, a lack of fundamental experimental knowledge about the crystal growth phenomena of **1a** is directly affected by the degree of supersaturation and supercooling states of **1a** on substrate surfaces, which may achieve the more flexible morphological control of crystals of **1a**.

Graphoepitaxy for organic materials is an approach to highly‐oriented thin films on amorphous substrates inducing anisotropic growth along with 1D physical barriers such as “walls” and “grooves” constructed on the surfaces.^[^ ^8^ ^]^ Ikeda et al. have reported the graphoepitaxy of a small molecular weight organic compound, *α*‐sexithiophene on the artificial periodic microgrooves of a thermally oxidized silicon surface by high vacuum deposition.^[^ ^8a,b^ ^]^ Wu and Feng et al. have demonstrated a guided physical vapor transport technique to control the growth, alignment, and positioning of single‐crystal wires of 9,10‐bis(phenylethynyl)anthracene on pillar‐structured silicon substrates.^[^ ^8c^ ^]^ Nevertheless, these graphoepitaxy approaches have never designated the crystallographic growth orientations without the help of anisotropic intermolecular interactions on source materials themselves.

Herein, we disclose an efficient fabrication method of the crystals of **1a** having specific morphologies by utilizing a patterning technique based on graphoepitaxy approaches. As the guidance of crystal growth of **1a**, convex lines having straight and curved shapes on glass substrates are successfully fabricated in cost‐effective procedures including spin‐coating with the positive resist and glass etching with hydrofluoric acid. Surprisingly, the alignments of micro‐droplets of **1a** along with such convex lines were observed in the initial stage of sublimation in the patterned growth process. Then, the appropriate surface temperature of the substrates and relative humidity are examined to generate the micro‐droplets of **1a** in the initial stage of sublimation. Finally, reversible photochromism in patterned crystals of **1a** has been demonstrated to highlight their potential applications and present an outlook for future opportunities.

## 2 Results and Discussion

### 2.1 Fabrication of Patterned Crystals

A precise control of positions where melts of **1a** can be transformed into crystals of **1a** on substrate surfaces is required to achieve the patterned growth on the graphoepitaxial substrates. First, substrate surfaces are adjusted to the appropriate temperature for the generation of the melts of **1a** there. In our previous works, a beaker containing ice water was put directly on the top substrate surface when heating from 30 to 98 °C with a hotplate. On the hydrophilic surface of substrates, the sublimate of **1a** was crystallized into fine crystallites after heating for 10 min. Whereas on the spherical substrates, a plastic bag containing ice water was put directly on the top substrate surface when starting the heating from 30 to 98 °C (Figure S1a, Supporting Information), the melt droplets of **1a** were generated at the center of their surfaces (Figure S1b,c, Supporting Information). Then, we tried measuring the surface temperatures of these substrates using commercially available thermosensor devices to estimate the proper surface temperature of the graphoepitaxial substrates. When measured after heating for 10 min, surface temperatures of the flat and spherical substrates were 28 and 13 °C, respectively (Figure S2a,b‐ii, Supporting Information). Here, calculated thermal gradients are defined as follows:

(1) $$\Delta T/\Delta Z=\left(T_{\mathrm{heat}}-T_{\mathrm{sur}}\right)\;/\;\left(Z_{\mathrm{sc}}+Z_{\mathrm{pd}}\right)$$

where *T*_heat_, *T*_sur_, *Z*_sc_, and *Z*_pd_ are the heating temperature with the hotplate (°C), the surface temperature of the substrate (°C), the height of the spherical cap (mm), and the height of the petri dish (mm). The calculated thermal gradients obtained from our measurements of surface temperatures of the flat and spherical substrates are shown in **Table** **1**. On the spherical substrates, the lower thermal gradient resulted in a lower degree of supersaturation than on the flat substrates. Thus, such insufficient supersaturations lead to the melt of accidentally generated interfaces of product nuclei there.

**Table 1:** Physical phases of **1a** generated on substrate surfaces.

Substrate	*ΔT*/*ΔZ* ^a)^ [°C mm^−1^]	Physical phase of **1a**	Refs.
Flat	4.7	Crystal	[6a]
Spherical	3.4	Liquid	[6b]
Flat	3.7	Liquid	This work

^a)^ *ΔT*: Difference of the surface temperature of the substrate from the heating temperature with the hotplate. *ΔZ*: Sum of the height of the spherical cap and the height of the petri dish.

**Figure 2:**
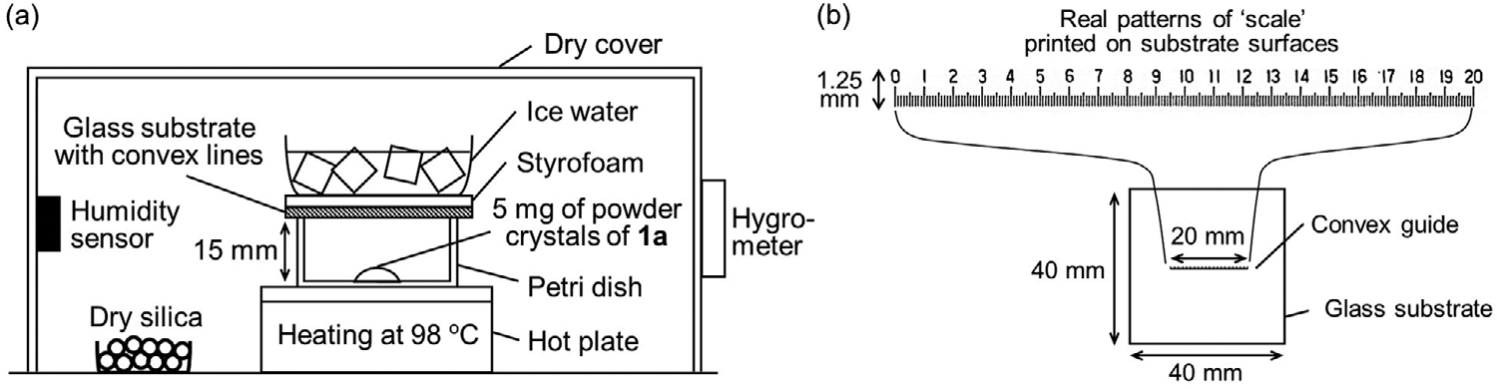
a) Sublimation setups used or graphoepitaxy of **1a** onto the glass substrate with convex lines under the control of relative humidity. The inner diameter of the petri dish is 32 mm. b) The real patterns of the convex lines fabricated on the substrate surfaces, which is 20 mm scale subdivided into 200 divisions of 0.1 mm printed on a glass scale. The surface temperature of substrates during the sublimation at 98 °C is adjusted to be higher than that for our previous work^[^ ^6a^ ^]^ by putting a sheet of Styrofoam between the beaker containing ice water and the glass substrate as shown in (a).

**Figure 3:**
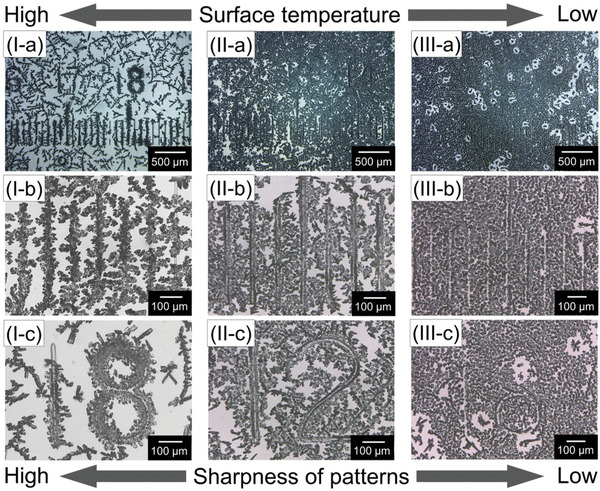
Different visualities of patterned crystals of **1a** generated on the respective region on the substrate surface having I) the relatively high, II) intermediate, III) relatively low temperatures. a) The higher the surface temperature at a certain place on the substrate, the more clearly the crystalline patterns of **1a** are formed there. The patterned crystals of **1a** on b) the straight convex lines arranged side by side and c) the curved convex lines having the shape of (I, III) ‘8’ and (II) ‘2.’ These different sharpness of patterns of **1a** on the substrate surface are observed reproductively for each sublimation.

In line with this preliminary knowledge, we adjust the highest surface temperature of the substrate for graphoepitaxy to be higher than that for our previous work by preparing the new setups as shown in **Figure** **2**a. The surface temperature rises to 42 °C by heating for 5 min after the precooling stage as shown in Figure S2c‐ii (Supporting Information). That surface temperature of the substrates may have been caused by a sheet of Styrofoam laid on the top face of the substrates and below a beaker containing ice water. In this case, the calculated thermal gradient is 3.7 °C mm^−1^, compared with 4.7 and 3.4 °C mm^−1^ at the center positions of substrate surfaces in our previous setups with the flat and spherical substrates, respectively. It is considered that the thermal gradient is low enough that the melts of **1a** can be generated on the substrate surfaces in the early stage of sublimation. Then we attempted to attain the patterned crystals of **1a** by employing the new sublimation setups. After heating for 1 h under the adjustment of relative humidity to 42 ± 3%, the patterned crystals of **1a** were generated on the convex lines with the different sharpness of patterns at each position within the same substrate surface as shown in **Figure** **3** and such patterned crystals of **1a** were reproducible. At one time, on top of the convex lines in the shape of the numerical characters, ranging from “0” through “20”, the microcrystals were gathered as shown in Figure S3 (Supporting Information). Also, it is presumed that generations of polycrystalline thin films having the same morphology as those generated on the flat substrates used in the sublimation of our previous works^[^ ^6a^ ^]^ was attributed to the fact that crystallization positions were accidentally the lower‐temperature area as shown in Figure 3III. It is also supposed that the crystals are easier to gather on the convex guides in the case where crystallization positions were the higher‐temperature area as shown in Figure 3I. Moreover, a lot of the melting drops are left at the edge of the surfaces of the substrate because of the heat transfer through the brim of the petri dish from the hotplate. This indicates that microcrystals are formed under the presence of their melts like the Ostwald ripening. We observed the fabrication process of the patterned crystals formed on the patterned convex lines.

### 2.2 Mechanism of Patterned Growth of Crystals

**Figure 4:**
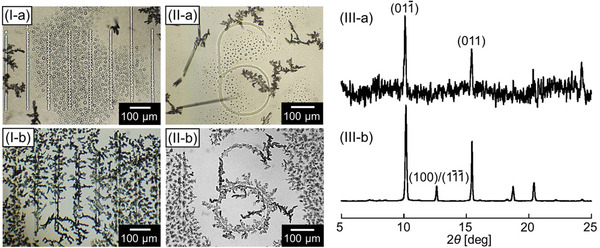
I, II) Optical microscopic images of sublimates and III) XRD patterns for substrate surfaces after the sublimation for a) 10 and b) 20 min. (I, II‐a) Micro‐droplets and (I, II‐b) microcrystals of **1a** (triclinic, *P‐*1, *a* = 8.833 Å, *b* = 11.178 Å, *c* = 11.431 Å, *α* = 100.588°, *β* = 112.708°, *γ* = 113.218°)^[^ ^9^ ^]^, have been generated on sidewalls of the convex lines on the respective substrates.

We characterized crystals generated on the substrates after different sublimation times. After heating for 10 min, many micro‐droplets of **1a** and a few microcrystals of **1a** have been generated as shown in **Figure** **4**I, II‐a. After heating for 20 min, microcrystals of **1a** have been generated on the side faces of the convex structures as shown in Figure 4I, II‐b. According to the X‐ray diffraction (XRD) measurements for microcrystals on either substrate surface after heating for 10 or 20 min, sharp peaks of the (01‐1) and (011) planes were detected with relatively high and low intensities, respectively, as shown in Figure 4III‐a,b.

To put our experimental results into schematic illustration as shown in Figure S4b (Supporting Information), there is an obvious difference between the crystal growth process on flat substrates for our previous work and this work. In the early stage of sublimation, the microdroplets of **1a** are generated on the substrate surface with that higher temperature. Then, the crystal faces of the (01‐1) planes, which tend to be formed on hydrophilic surfaces, are produced but they never grow into the large crystalline domains, like the polycrystalline thin films, because of the presence of microdroplets. Therefore, the proportion of major Miller indices for the top faces of microcrystals of **1a**, including the (01‐1) and (011) planes were kept in the crystallization process of microdroplets of **1a**.

In addition, the patterned crystals of **1a** were never observed on the substrates at certain ranges of relative humidity. When the sublimation is conducted at more than 45% of relative humidity, polycrystalline thin films of **1a** are formed on the substrate surfaces because of the increase in surface energies of substrate surfaces, stemming from the adsorption of moisture. As shown in Figure S5I‐a (Supporting Information), there are a large number of small possible areas of nucleation as presented in grey circles at the high densities. As a result, tightly packed microcrystals of **1a** are generated both on the convex lines and the other surface spaces as shown in Figure S5II,III‐a (Supporting Information). According to microscopic observations of these microcrystals under crossed polarizers, the rhombus‐shaped crystals, whose top faces are the (01‐1) planes, have been generated with ordered in‐plane orientations as shown in Figure S7d (Supporting Information). Whereas, at less than 39% of relative humidity, a large amount of liquids of **1a** were produced on substrate surfaces due to the decrease in surface energies of substrate surfaces. In such cases, there are a small number of large possible areas of nucleation at low densities as shown in Figure S5I‐c (Supporting Information). The rhombus‐shaped crystal faces are expanded and branched in the directions of the [01‐1] and [100] axes at a fast rate^[^ ^6b^ ^]^ until the crystal faces reach within any of the possible areas of nucleation. These large‐sized liquids of **1a** crossed over the convex lines and prohibited the generations of patterned crystals of **1a** as shown in Figure S5II,III‐c (Supporting Information). At the intermediate relative humidity, the rhombus‐shaped crystal faces formed in a specific possible area of nucleation can simultaneously boost the possibilities of nucleation in the adjacent possible areas of nucleation through their rearrangements as shown in Figure S5I‐b (Supporting Information). Therefore, the microcrystals were produced with highly‐ordered in‐plane orientations as shown in Figure S5II,III‐b (Supporting Information). In summary, the relative humidity can affect the frequency of nucleation events of **1a**, then the patterned growth was changed depending on the relative humidity. The effect of the substrate surface temperature on the in‐plane orientations will be discussed in more detail in the following section.

### 2.3 Growth Orientations of Patterned Crystals

**Figure 5:**
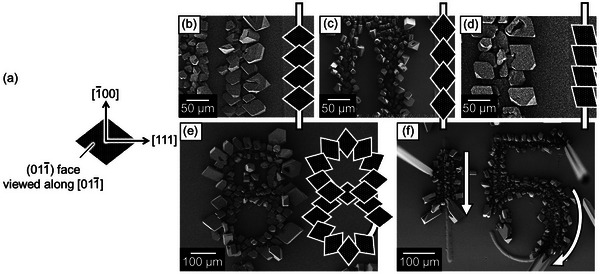
a) Schematic illustrations of crystallographic orientations of the rhombus‐shaped (01‐1) faces viewed along [01‐1], which depict ones for the same shaped figures on the left sides of SEM images in b–e) at the same time. (b–f) SEM images of microcrystals of **1a** patterned on each (b–d) straight and e,f) curved convex lines. In‐plane orientations of rhombus‐shaped (01‐1) faces aligned on convex straight lines are classified into three different types as shown in (b–d). (e) Specific radial in‐plane orientations of such crystal faces with respect to the curved lines shaped the numeric number of ‘8’. f) Patterned crystals have been yielded without any gaps on the convex straight and curved guides shaped by the numeric number “5”. The white arrows indicate in‐plane crystal growth directions along the straight and curved convex guides.

In‐plane crystallographic growth orientations of microcrystals of **1a** included in the patterned crystals are observed by scanning electron microscopy (SEM) as shown in **Figure** **5**. The three kinds of in‐plane crystallographic growth orientations of rhombus‐shaped crystal faces of the (01‐1) planes (Figure 5a) are mainly generated on straight lines as shown in Figure 5b–d. Such uniformity of orientations of the rhombus‐shaped crystal faces on the convex straight lines can be attained through the graphoepitaxial growth process under the presence of the liquid phases of **1a**.

This phenomenon resembles the evaporation of the water droplets on the heated sheets of aluminum foil, having the hairline texture on its surface.^[^ ^10^ ^]^ Each 1 µL of water droplet put on the surface of respective sheets of aluminum foil, whose surface temperatures were adjusted to 20 and 40 °C, was exhausted isotropically and anisotropically, respectively (Figure S6a,b, Supporting Information). Such evaporation events indicate that when the liquid has low surface energies for the solid surface, the droplets are evaporated anisotropically along with the 1D physical barriers like the hairlines on a sheet of aluminum foil. In the case of the aligned droplets of **1a** on the sidewalls of the convex lines, the anisotropic evaporations of these droplets are triggered by heat flows parallel to such convex guides. Then, Alignments of the droplets of **1a** take on directional and consecutive crystallization events, which causes the chains of the rhombus‐shaped crystal faces with the same crystallographic growth directions.

Regarding the control factor of in‐plane orientations of microcrystals generated on the straight convex lines, it is considered that each in‐plane orientation of rhombus‐shaped microcrystals, whose top faces have the (01‐1) planes, are determined by directions of heat gas flows of **1a** just above substrate surfaces as referred in Figure S6c (Supporting Information).

At the same time, these SEM images show that the size of the crystals being formed ranges from 5 to 50 µm. The size of the microcrystals of **1a** aligned on convex guides depends on how the crystallization positions are distant from the convex lines. The small‐sized crystals have been generated at very close positions to the convex lines, whereas the large‐sized crystals have been yielded at distant positions from the convex lines. Particularly, the small‐sized crystals produced at the inner fringe of the convex guides shaped the numeric number of “8”, whereas the large‐sized crystals were produced at the outer fringe of them. This is also because heat gas flows of **1a** can go into outside positions of them instead of the inside ones.

### 2.4 Summary of Advances in Developed Patterning Methods

The patterning through the mechanism found in this work is distinguished from that of conventional patterning utilizing graphoepitaxy for fabrications of the single crystalline thin films of organics. As for conventional graphoepitaxy, high vacuum conditions, leading to the surface‐temperature‐dependent degree‐of‐supersaturations, are essential to achieve the goal of getting the highly ordered in‐plane orientations for thin films through self‐assembly of molecules in nanometer periodic grooves. Under such a control of the degree of supersaturation, at the same time, the increased nucleation rate can be easily accomplished by surface wettability and temperature of substrates. It means, however, that polycrystalline thin films, having disordered in‐plane orientations, are easy to form due to the increase in the number of points of origin of crystal growth on substrate surfaces as mentioned by Ikeda et al.^[^ ^8a^ ^]^ Therefore, the key factor for achieving the conventional graphoepitaxy is the use of source materials with the potential to form highly anisotropic crystal packings inferred from the Bravais–Friedel–Donnay–Harker laws,^[^ ^11^ ^]^ which allow the materials to undergo the 2D thin‐film growth under the desired supersaturation conditions. Considering the many respects mentioned above, the scientific novelty and technological advances in patterning methods of the present work are as follows:

1. Patterning along with the ordered in‐plane orientations through graphoepitaxy, for the first time, has been accomplished despite the use of a compound that forms the specific crystal packing, comprising the similar lattice dimensions and angle of a unit cell, which never helps the anisotropic growth in the specific crystallographic orientations.
2. Such patterning methods, achieved under the atmospheric pressure condition through a vapor‐to‐liquid‐to‐crystal process, will be used for a wide range of low‐molecular‐weight organic compounds as well as **1a**.

### 2.5 Photochromism of Patterned Crystals

**Figure 6:**
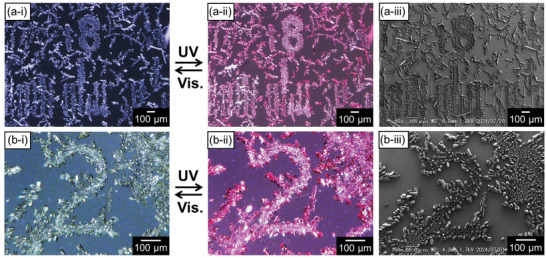
Reversible photochromism of patterned crystals of **1a** is demonstrated by alternating irradiation with UV and visible light. Optical microscopic images of the patterned crystals of **1a** after irradiations with a,b‐i) visible and (a,b‐ii) UV light. (a,b‐iii) SEM images of crystal morphologies of those patterned crystals. The crystalline patterns fringed with the rod crystals are clearly colored in red upon irradiation with the UV lights as shown in (a,b‐ii).

After the alternative irradiations with UV and visible lights for the substrate surface, the patterned crystals of **1a** can exhibit the photo‐reversible photochromism in red as shown in **Figure** **6**a i, ii,6b‐i, ii. At the same time, the crystals on top of the convex guide are smaller than the rod crystals around the convex guides (Figure 6a,b‐iii), and the former are colored in lighter red than the latter. Therefore, it seems that each numeric character is fringed with micro‐rod crystals under the irradiation with UV light. According to polarized absorption spectra for the single crystalline phases with the crystal faces of the (01‐1) and (100)/(1‐1‐1) planes, the intensity of the color of the former was weaker than that of the latter.^[^ ^9^ ^]^ Considering the contrast of depth in red color depending on the crystal morphologies of microcrystals of **1a**, these microcrystals of **1a** located on top of and around the convex lines have the crystal faces of the (01‐1) and (100)/(1‐1‐1) planes in their top surfaces, respectively. The reversible photochromism in these patterned crystals of **1a** suggests the utilities as photomechanical materials after they can be detached from the glass substrate.

As for an outlook of coupling the patterning and photochromism of organic crystals, it is possible to form a surface that changes its wettability before and after light irradiation as a flow path, as has been achieved in several previous studies. In the case of **1a**, however, the photo‐switching of surface wettability was not observed for its crystalline surfaces. In this respect, we should explore another suitable diarylethene derivative in the future.

Furthermore, the crystalline sublimates on the graphoepitaxial substrates can perhaps grow up to the centimeter‐sized patterned crystals. In brief, our sublimation methods are one of the promising ways of fabricating large‐sized photomechanical materials.

## 3 Conclusion

We have developed a new patterning method through graphoepitaxy, based on the crystal growth properties of **1a** in the vapor phase under the precisely controlled thermodynamic condition. In the early stage of the sublimation of **1a**, plenty of the micro‐droplets of **1a** were generated on the sidewalls of convex lines having straight and curved shapes under adjustments of the surface temperature of substrates. These micro‐droplets were crystallized into the chains of rhombus‐shaped microcrystals of **1a**. Each 1D alignment of the microcrystals has highly‐ordered in‐plane crystallographic orientations due to the anisotropic evaporation events for the micro‐droplets of **1a** along the convex guides. The patterned crystals of **1a** on the convex guides are fringed with micro‐rod crystals whose side faces have higher absorption intensities, therefore such patterned crystals have clear visibility under irradiation with UV light. In addition, the patterning method in this work has the unique advantage in morphological control over crystal‐growth methods in our previous works^[^ ^6^ ^]^ that is the “as‐depicted” morphological control for crystals of **1a**, achieved through the crystallization at selective positions controlled in the preparation phase of the convex guides onto substrates. We believe this patterning technique will enhance the understanding of the relationship between the crystal morphology and the photomechanical property by providing diverse crystal morphologies for photomechanical crystalline materials in the future.

## 4 Experimental Section

### Pretreatment of Surfaces of Glass Substrate

A glass substrate was immersed in a mixed solvent of methanol and hydrochloric acid (1:1) (30 min), thoroughly washed with distilled water, dried under reduced pressure, then immersed in concentrated sulfuric acid for 24 h, thoroughly washed with boiling distilled water, and then vacuum‐dried. The wettability of the surface of the prepared glass substrate was confirmed by measuring the contact angles (*θ*) of the water drop (1 µL) on it (*θ* = 8°). The clean glass plate was used as a substrate with a hydrophilic surface. The glass substrate was cleaned as described above, and was treated with *n*‐hexyltrimethoxysilane (*n*‐HTMS) by the wet method as follows: the water/ethanol solution was adjusted (pH 3.3) using acetic acid and stirred (10 min, 25 °C). Then, *n*‐HTMS (2 wt.% relative to the water/ethanol solution) was added to the stirred mixture and stirred subsequently (5 min). The preprocessed glass substrate was soaked in the mixture (2 min). After that, the substrate was rinsed with ethanol and dried in a vacuum (110 °C, 5 min). The contact angle on the hydrophobic surface of the prepared glass substrate was 102°.

### Photoresist and Pattern‐Transfer Process

The convex guides onto the surfaces of glass substrates were fabricated following the steps, as shown in Figure S8a (Supporting Information). The glass substrates having the hydrophobic surface were spin‐coated with commercially available positive photoresists (FPPR‐P30ET, Fuji Chemicals Industrial Co., Ltd.). First, the glass substrate was put on a sample holder of a spin coater apparatus and photoresist solutions (2 mL) were dispensed on the surface of the glass substrate. The spin coater was rotated (2000 rpm, 15 s) and the glass substrate with a photoresist layer was prebaked (120 °C, 2 min). The surface of a glass scale printed with a 20 mm scale subdivided into 200 divisions of 0.01 mm was contacted to the surface of the spin‐coated substrate, and 365 nm UV light was exposed (2 min) over the glass scale by the handy lamp (SLUV‐4, AS ONE) to transfer such patterns of “scale” onto the substrate surface coated with the photoresist film.

### Development of Patterns

Tetramethyl ammonium hydroxide (TMAH) methanol solution (10 wt.%) was diluted with distilled water to prepare TMAH solutions (1.19 wt.%). The glass substrate coated with photoresist was put on the base of a beaker and its top face was puddled (5 s) by the diluted TMAH solutions (1.5 mL), and then left to stand in the dark (1 min). The glass substrate was washed with a large amount of distilled water and dried on the hot plate (120 °C, 1 min).

### Etching of Substrate Surfaces

After the development process, the substrates were soaked (3 min) in hydrofluoric acid solution (15 wt.%, 50 mL), then washed with a large amount of distilled water and dried on the hot plate (120 °C, 1 min). Finally, the unremoved photoresist films on the substrate surfaces were removed thoroughly with acetone. The convex guides as shown in Figure S8b‐i,ii (Supporting Information) have been fabricated through these steps according to observations of these convex guides using a Keyence color 3D laser scanning microscope VK‐8700/8710 and its observation application VK‐H1V1. Their side view profile showed that the shape of prepared convex guides is shaped like a summit, whose bottom line are lower than the horizontal lines of the flat portions of substrates, as represented in Figure S8b‐iii (Supporting Information).

### Adjustment of Relative Humidity Before Heating

To adjust the humidity conditions in a sublimation system, the whole of the sublimation apparatus was covered with a dry cover equipped with a thermometer and a hygrometer (BLE‐SD12‐010, Saginomiya Seisakusho, Inc). A certain relative humidity inside a dry cover (25%) was kept in the use of Silica Gel until the next stage of pre‐cooling. A certain temperature of the hotplate (30 °C) was maintained until the next stage of pre‐cooling. A certain temperature of air conditioning (21 °C) was set and “dry” mode. The glass Petri dish (height: 15 mm, inner diameter: 32 mm) containing powder crystals of **1a** was put on the hotplate inside the dry cover at the adjusted relative humidity and the pre‐cooling was started when a 200 mL glass beaker containing ice water was put on the upper surface of the guide substrate. The glass beaker with the flat base should be used so that distributions of the surface temperature of the guide substrate are the same in every experiment. The relative humidity was adjusted to gradually increase (25–31%) during the pre‐cooling time and the petri dish was heated (98 °C) on the hotplate at the end of the pre‐cooling time. At the same time, the beaker was put aside during the first 5 min of heating.

### Adjustment of Relative Humidity After Heating

The moisture was added inside the dry cover so that the relative humidity reached a certain relative humidity (42%) with a mist at the beginning of heating (within 5 min). After the first heating stage (5 min), the guide substrate was cooled with a beaker containing ice water put onto a sheet of Styrofoam (area: 40 mm square, thickness: 2 mm) with a round hole (diameter: 32 mm) in the center, laid on the upper surface of the guide substrate. During the sublimation (1 h), the temperature and humidity inside the dry cover were recorded and replenished the ice water at regular intervals (5 and 30 min, respectively).

### Measurement and Definition of Surface Temperature of Substrates

Kapton film thermo‐sensor (TJK‐SCP07FP, Jsensor) connected with a data logger (HJ‐UDL‐TC, SATOTECH) was adhered to the surface of the glass substrate and the glass petri dish was covered with the glass substrate, whose bottom surface was attached with Kapton tape, then the petri dish was put on the hot plate at a certain surface temperature (30 °C). The surface temperature of the bottom surface of the glass substrate was measured by the data logger. Differential scanning calorimetry (DSC) was performed for **1a** using a Hitachi DSC7000X instrument.

### Characterization of Patterned Crystals

To determine miller indices of the top faces of microcrystals, a benchtop powder XRD instrument (MiniFlex, Rigaku) was employed. The glass substrate with microcrystals on convex guides was set in the sample holder in the XRD instrument.

Before starting the observation by SEM device (VE‐7800, KEYENCE), the glass substrates with microcrystals on convex guides were sputter coated with an ultrathin layer of gold by vacuum vapor deposition equipment (MSP‐1SK, Vacuum Device Inc.). Then, the glass substrates were fixed on metallic sample holders with cut carbon tapes.

### Observation of Photochromism of Patterned Crystals

The microcrystals on glass substrates were observed using a Nikon ECLIPSE E600POL polarizing optical microscope. Visible light irradiation was performed using a halogen lamp (100 W). Photochromism of microcrystals was observed using a Keyence VHX‐500 digital microscope. UV irradiation was carried out using a Keyence UV‐LED UV‐400/UV‐50H (365 nm light). Visible light irradiation was performed using a halogen lamp (100 W).

## Conflict of Interest

The authors declare no conflict of interest.

## Supporting information



Supporting Information

## Data Availability

The data that support the findings of this study are openly available in ChemRxiv at 10.26434/chemrxiv‐2024‐j0sdf, reference number 1.
