# Supplementary material for: Patterning of Photochromic Diarylethene Crystals by Sublimation for Morphological Controls
Source: Small Methods. 2025 Jan 19;9(3):2401545. doi: 10.1002/smtd.202401545 (PMC11926499; doi:10.1002/smtd.202401545)
Supplement: Supplementary file 1 — Supporting Information [file SMTD-9-2401545-s001.docx]

Supporting Information

Patterning of photochromic diarylethene crystals by sublimation for morphological controls

Mami Isobe, Daichi Kitagawa, and Seiya Kobatake*

**Table of Contents**

I. Melt generated on surfaces of the spherical substrate ………………………………..….. S2

II. Surface temperatures of substrates for sublimation setups ………………………….….. S3

III. Clear patterning of crystals in the wide range …………………………………………. S4

IV. Sublimates on substrate surfaces with different temperatures ….……………….…….. S5

V. Nucleation densities and alignments of rhombus-shaped crystal faces ………….…….. S6

VI. Alignments of in-plane orientations of rhombus-shaped crystal faces ……….……….. S7

VII. Orientations of microcrystals under observations under crossed polarizers…….……. S8

VIII. Fabrications of convex guides on substrate surface ……………………......….…….. S9

**I. Melt generated on surfaces of the spherical substrate**

In the sublimation, the degree of supersaturation on substrate surfaces becomes lower to the larger distance between the substrate surfaces and the sublimation source. Such lower supersaturation can decrease the frequency of nucleation and promote the generation of liquid phases. In the case of the sublimation of **1a** onto the surface of spherical substrates, the droplets of melt of **1a** tend to be the lower supersaturation of **1a** in the center positions of the substrate surface, which is more distant from the powder crystals of **1a**. Therefore, the droplets of melt of **1a** are yielded there.


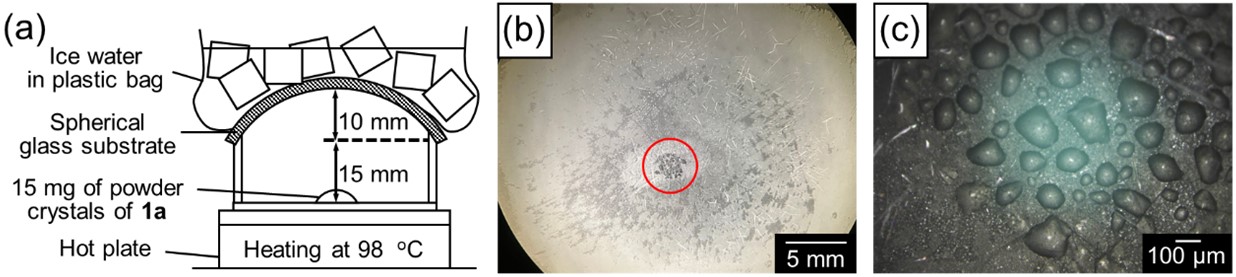


**Figure S1.** After the sublimation of **1a** is performed onto the ice-cooled surface of spherical substrates at 98 °C for 2 h as shown in (a), the products are confirmed by using the optical microscopy. (b) Polycrystalline thin films of **1a** are generated on the entire surface and the liquid phases of **1a** are yielded in its center positions (inside the red circle). (c) The magnified photograph of such regions shows multiple melt droplets of **1a**.

**II. Surface temperatures of substrates for sublimation setups**

The way the surface temperature of substrates changes during the sublimations is different from sublimation setups. The physical phases of sublimates and the crystal faces attached on substrate surfaces in the early stage of sublimations of **1a** are affected by the degree of supersaturation, which can be associated with the difference between the surface temperature of substrates and the heating temperature. The thermal gradients in Table 1 were calculated from the highest surface temperatures of the substrates measured for each sublimation setup. The surface temperatures of the flat and spherical substrates in our previous works rise to 28 and 13 °C after heating for 12 and 7 min with the hotplate, respectively. The surface temperature of the substrate in this work reaches 42 °C after heating for 5 min.

Furthermore, the maximum surface temperature of 42 °C, which allows the clear patterning of **1a** to be reproduced, was several degrees Celsius higher than the crystallization temperature of **1a** according to the heating curve in differential scanning calorimetry (DSC) measurements of **1a** as shown in Figure S2d. This suggests that the time-shifting of the surface temperature of substrates should be controlled so that sublimates can undergo supercooling states at the early stage of sublimation to reproduce the patterned crystalline phase.

**
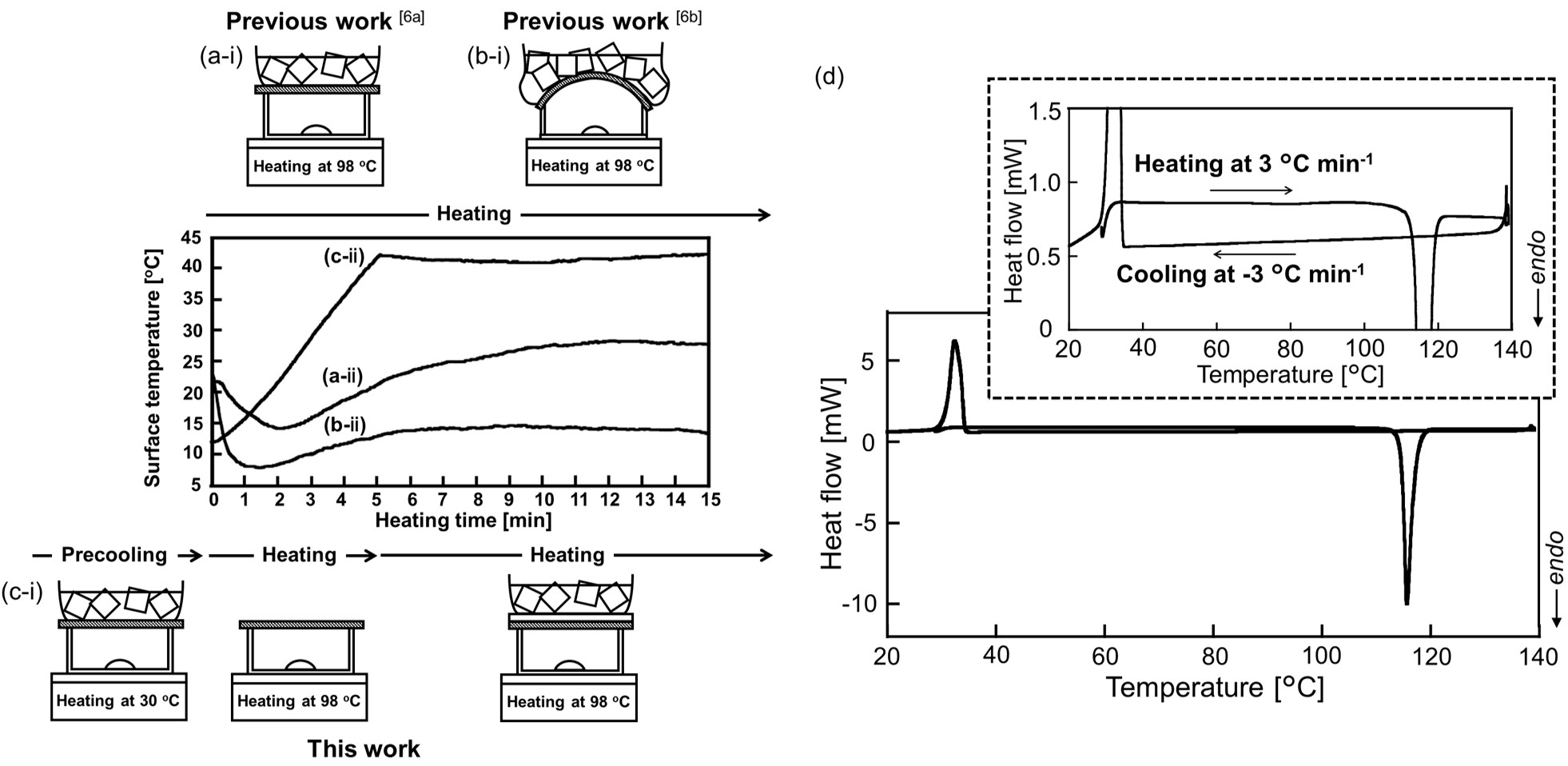
**

**Figure S2.** (a-ⅰ) The flat and (b-ⅰ) spherical substrates employed for the setups in the previous works and (c-ⅰ) the flat substrates used in this work. (a, b, c-ⅱ) Surface temperatures of the center position of each substrate surface measured while heating for 15 min at 98 °C. (d) DSC profile of **1a** in heating process from 30 to 140 °C at 3 °C min^−1^ and the following cooling process from 140 to 10 °C at −3 °C min^−1^. The figure inside the dotted square frame represents enlarged profiles of each DSC curve in the heating and cooling process including melting and crystallization peaks.

**III. Clear patterning of crystals in a wide range**

Patterning of crystals of **1a** can be produced in a wide range on the substrate surface.


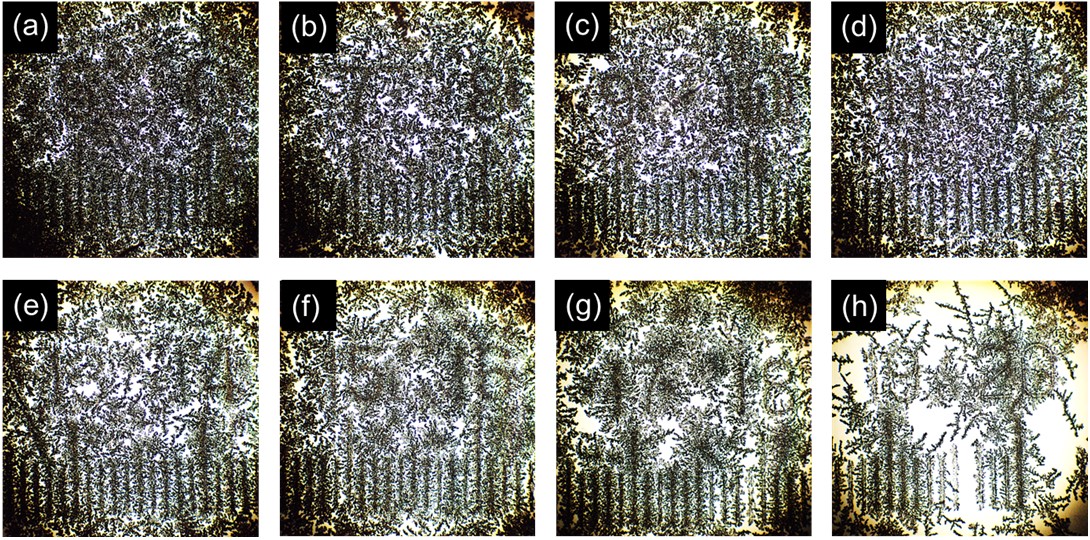


**Figure S3.** Photographs of the patterning of crystals of **1a** indicating the numerical characters such as (a) “5”, “6”, (b) “7”, “8”, (c) “9”, “10”, (d) “11”, “12”, (e) “13”, “14”, (f) “15”, “16”, (g) “17”, “18”, (h) “19”, “20” and the scale lines.

**IV. Sublimates on substrate surfaces with different temperatures**

The surface temperature of the substrates influences the physical phase of the sublimates and their attachments and expansions in the sublimation process. At the same time, the crystal faces of the crystalline sublimates can be identified from the XRD patterns for substrate surfaces after each heating time.

In our previous work, microcrystals are generated on the substrate surfaces with the lower temperature in the early stage of the sublimation, and the polycrystalline thin films having the (011) planes are formed as shown in (a). Then, the rod crystals of **1a** having the (01) planes are generated on these thin films at high density.

In this work, however, the multiple micro-droplets of melts of **1a** are attached on the substrate surface with a higher temperature in the early stage of the sublimation as shown in (b). At the same time, the microcrystals of **1a** to be generated at a low density instead of the expansion of the thin films on the substrate surfaces. Only a few of the rod crystals are generated on such grown microcrystals. Therefore, the proportion of Miller indices for the top faces of microcrystals of **1a**, the (01) and (011) planes, was kept in the crystallization process of micro-droplets of **1a** as shown in XRD measurements for sublimates after the different heating time (Figure 4Ⅲ).


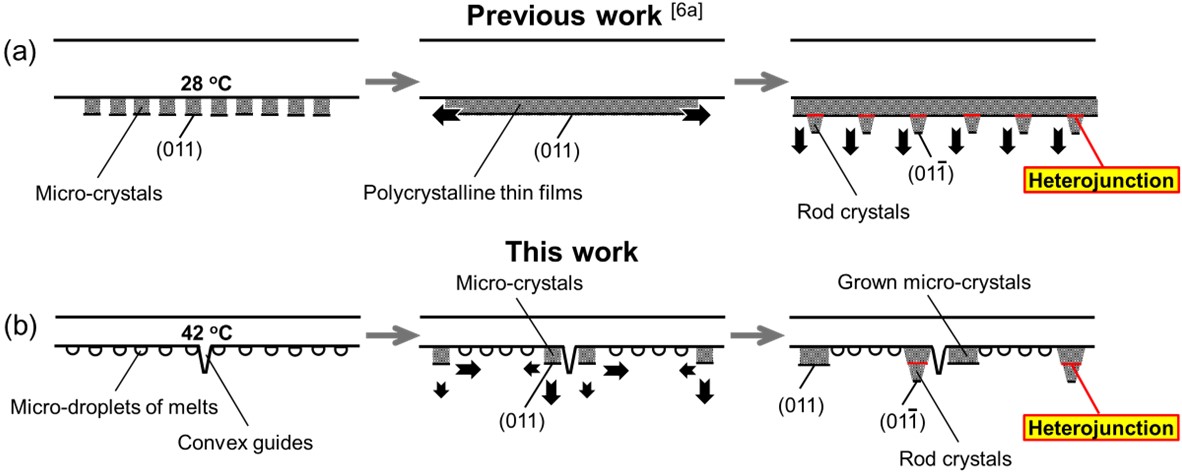


**Figure S4.** On substrates having the (a) lower and (b) higher surface temperatures can the higher and lower supersaturations of **1a** on the substrate surfaces, respectively. This causes the generation of different physical phases of **1a**, its nucleation at different densities, and the expansion of the respective crystal faces of **1a** at different proportions. The black arrows indicate the crystal growth directions of the respective kinds of crystal faces.

**V. Nucleation densities and alignments of rhombus-shaped crystal faces**

The nucleation densities of **1a** can be associated with the alignments of rhombus-shaped crystal faces of **1a**, the (01) planes. In the case of the higher nucleation density as shown in (I-a), the small-sized crystallites are generated at random in-plane orientations as shown in (II, III-a). Whereas in the case of lower nucleation density as shown in (I-c), the crystal faces grow into large-sized crystallites through the dendritic growth in the different growth directions along with the substrate surface as shown in (II, III-c). In the case of the intermediate degree of nucleation density, the crystallites generated with certain in-plane orientations can promote the generations of the adjacent crystallites with the same in-plane orientations one after another as shown in (I-b). Due to such a consecutive nucleation process, the alignments of crystal faces with the same in-plane orientations can be formed along with the convex guides as shown in (II, III-b).

**
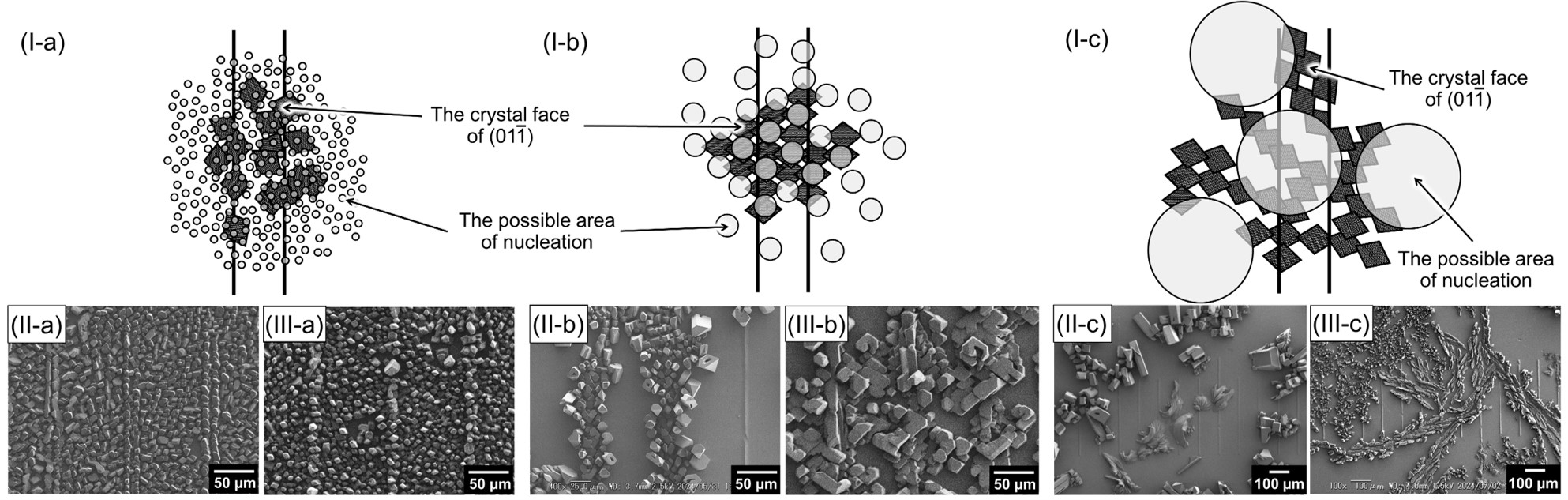
**

**Figure S5.** (I) Schematic illustrations and (II, III) SEM images for the relationship between nucleation densities of **1a** and alignments of rhombus-shaped crystals of **1a** under the (a) higher, (b) intermediate, and (c) lower supersaturations: where the grey circles indicate the possible area of nucleation of **1a** and the black rhombus figures presents the crystal faces of the (01) planes of **1a**. (II, III-a) The rhombus-shaped microcrystals of **1a** having the random in-plane orientations are generated at high densities. (II, III-b) The alignments of rhombus-shaped microcrystals of **1a** have taken on the highly-ordered in-plane orientations by the rearrangements of the possible areas of nucleation of **1a** at the densities, which may determine the following nucleation positions. (II, III-c) The melt phases of **1a** undergo the rapid growth along with the [111] and [00] axes to produce the dendritic crystals of **1a**.

**VI. Alignments of in-plane orientations of rhombus-shaped crystal faces**

Chains of rhombus-shaped microcrystals of **1a** having specific in-plane orientations are generated under the successive evaporations of melt drops of **1a** along with the sidewalls of the convex guides. This mechanism is supported by the evaporation phenomena of water droplets in parallel directions to hairlines on surfaces of sheets of aluminum foil. Once water droplets were put on the surfaces of the aluminum sheets at 20 and 40 °C, their top faces shrank isotropically (a) and anisotropically (b) during the evaporations, respectively. In short, the water droplets having higher wettability to the surface of aluminum foil tend to drain at faster speeds in the parallel directions to the hairlines than the vertical directions to them. The respective kinds of the in-plane orientations of the rhombus-shaped crystal faces of **1a** observed on the convex lines depend on the direction of the heat gas flows of **1a** to the convex lines as shown in the red arrows in (c).


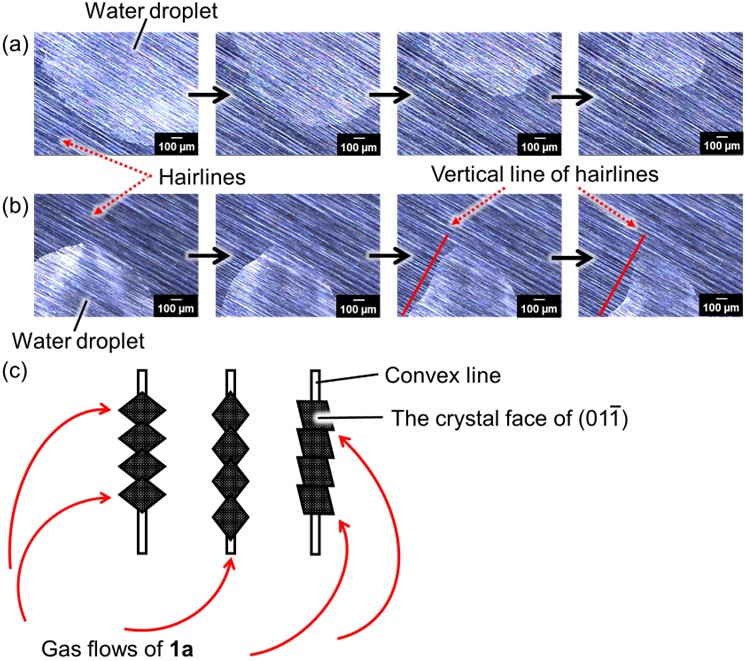


**Figure S6.** The water droplet has lower and higher wettability on the surfaces of such sheets adjusted to (a) 20 and (b) 40 °C, respectively. (a) When 1 µL of water droplets were put on the surfaces at 20 °C, the water droplet was evaporated isotropically. (b) On the surface of 40 °C, the evaporations along with the hairlines occur at a faster rate. (c) The respective kinds of crystallographic growth orientations with respect to the convex lines can be determined by the traveling directions of gas flows of **1a** presented by the red arrows, affecting the crystallographic directions of rhombus-shaped crystal face of **1a** on the convex guides.

**VII. Orientations of microcrystals** **under observations under** **crossed polarizers**

Optical microscopy images of microcrystals of 1a on (a-d) the convex straight lines and (e, f) the convex guides with the shape of numerical number under (ⅰ) the natural lights and (ⅱ) crossed polarizers are as shown in Figure S7. The in-plane orientations of these microcrystals can be characterized by crystallization area within striped convex straight lines, the size of microcrystals yielded on the convex lines, and the distance from the convex lines. Each photograph depicts these characteristics as follows:

(a-f): The smaller size the microcrystals generated on each of the convex guides have, the brighter appearance the entire crystalline pattern can exhibit.

(a, b): The brighter and darker areas appear not for each of a single convex line but for each area over multiple lines because the air currents cause crystals of the same orientation to be aligned on the convex straight lines that are close to each other.

(c, d): The small-sized crystals generated very close to the convex lines have the same in-plane orientation, resulting in the same brightness of these crystals.

(e, f): Regarding the microcrystals generated on the convex guides having the shape of “8”, they have different in-plane orientations with respect to the directions of the polarizer set for the optical microscopy. As a result, some of these microcrystals can look bright and others look dark under the crossed polarizers.

**VIII. Fabrications of convex guides on substrate surface**


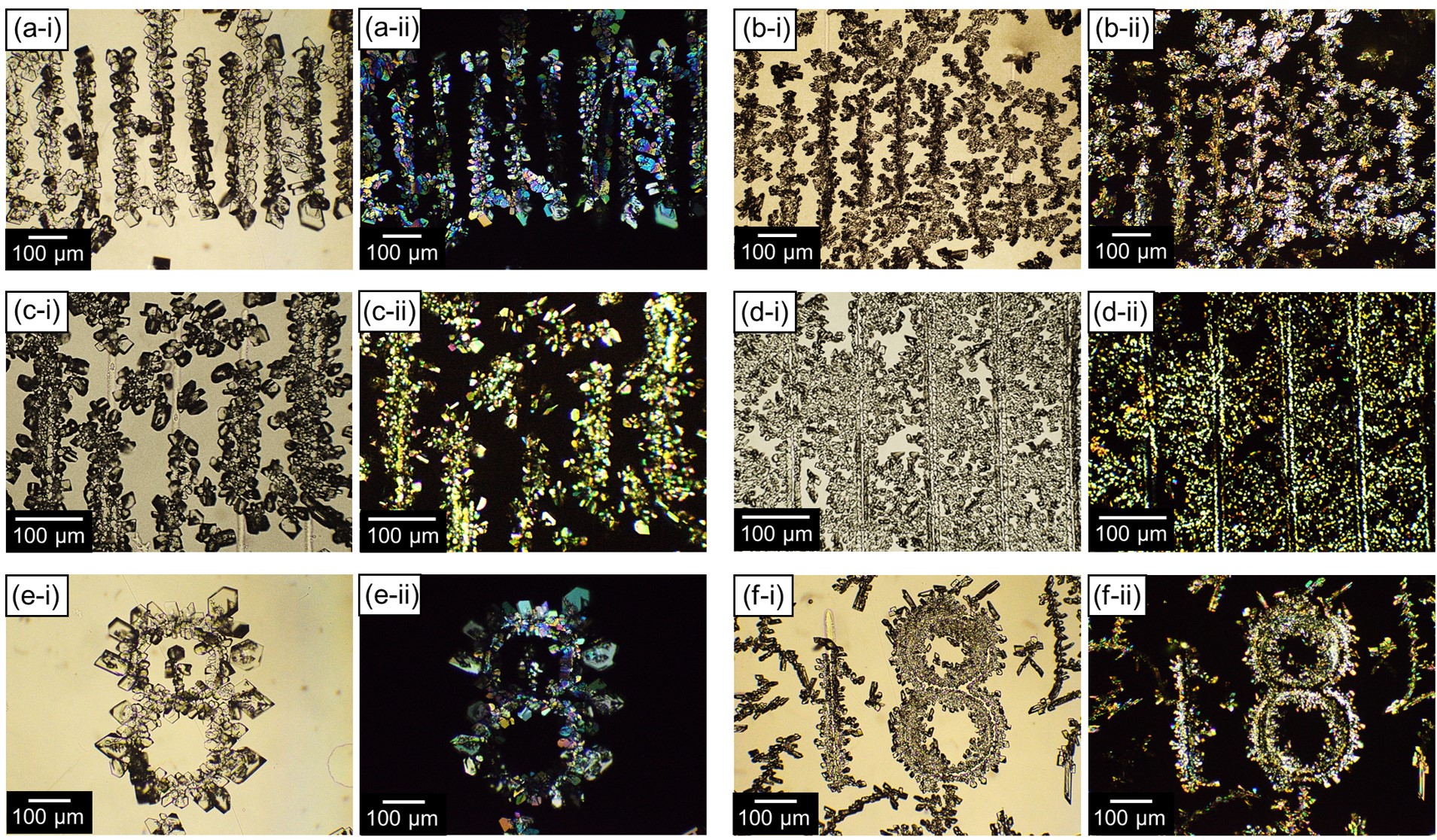


Figure S7. Optical microscopy images of microcrystals of 1a on (a-d) the convex straight lines and (e, f) the convex guides with the shape of numerical number under (ⅰ) the natural lights and (ⅱ) crossed polarizers. In each polarizing light microscopic photographs, the polarizer and analyzer of the polarizing microscope are oriented in the lateral and longitudinal directions, respectively.

The process for the glass substrate surface has been examined to fabricate the convex guides with the proper height and width to achieve the graphoepitaxy of **1a**. The height and width of the convex guide in the shape of the summits highly depend on the etching time and the concentration of etching solutions.

**
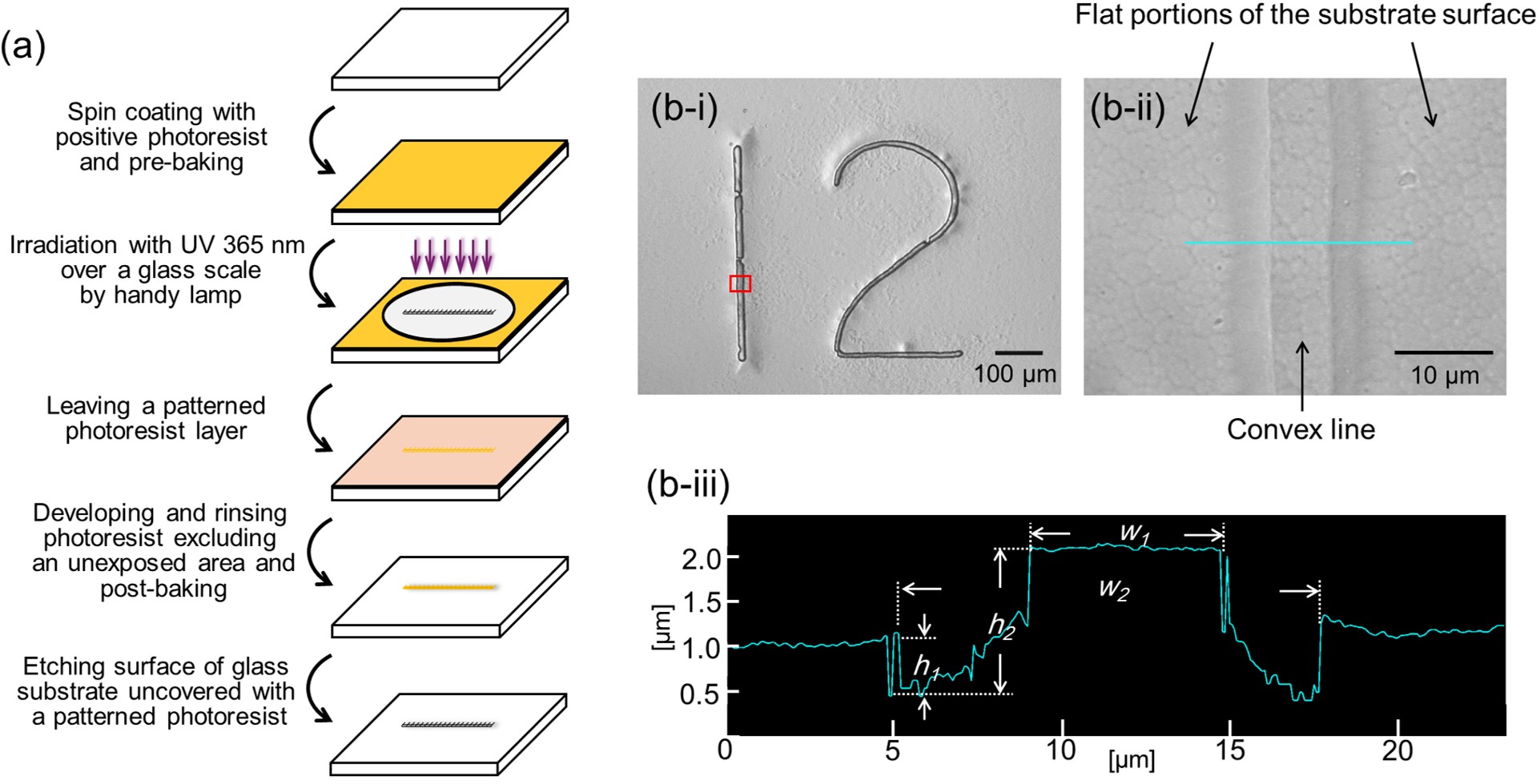
**

**Figure S8.** (a) Fabrication procedure of the convex lines on the surface of the glass substrate through spin-coating with positive photoresist and glass etching with hydrofluoric acid. The surface roughness of the glass substrates after the etching process is measured by using confocal laser microscopy. (b-ⅰ) The photographs indicate top views of the convex line in the shape of “12” constructed on the substrate surface, (b-ⅱ) the magnified images of the parts inside the red square frame in (b-ⅰ). The side view profile for the light blue line as shown in (b-ⅲ) shows that prepared convex guides are shaped like a summit whose top and bottom faces are 6 (*w*_1_) and 12.5 (*w*_2_) µm wide, respectively. The distance between the bottom face of the summit and the flat surface of the substrate surface is 0.5 (*h*_1_) µm and the height of the summit is 1.5 (*h*_2_) µm.
